# Rapidly growing and ulcerating metastatic renal cell carcinoma of the lower lip: A case report and review of the literature

**DOI:** 10.3892/ol.2014.2505

**Published:** 2014-09-05

**Authors:** JUHO SUOJANEN, ESA FÄRKKILÄ, TESSA HELKAMAA, VENLA LOIMU, JYRKI TÖRNWALL, CHRISTIAN LINDQVIST, JAANA HAGSTRÖM, KARRI MESIMÄKI

**Affiliations:** 1 Department of Oral and Maxillofacial Diseases, Helsinki University Central Hospital, Helsinki FIN-00014, Finland; 2 Department of Oncology, Helsinki University Central Hospital, Helsinki FIN-00014, Finland; 3 The Haartman Institute, Department of Pathology and HUSLAB, Helsinki University Central Hospital, Helsinki FIN-00014, Finland

**Keywords:** renal cell carcinoma, head and neck, metastasis, lip

## Abstract

Renal cell carcinomas (RCCs) have a tendency to metastasize at an early stage, therefore, the patients frequently exhibit metastatic disease at the time of diagnosis. Common locations for the metastases are adjacent organs and abdominal lymph nodes; however, occasionally metastasis to the peripheral organs may be the initial clinical symptom. The 71-year-old male patient in the current case suffered from radioresistant and aggressively behaving RCC metastasis in the mandible and lower lip, which was successfully managed by surgical resection. RCC metastasis to the facial area is considered to be uncommon based on a review of the existing literature. RCC are somewhat radioresistant and therefore, palliative surgery must be considered when treating patients with this metastatic disease.

## Introduction

Renal cell carcinoma (RCC) is divided into clear-cell, papillary, oncocytoma and collecting duct subtypes which exert different invasion and metastatic potentials (1). The clear-cell carcinoma subtype represents <85% of reported cases, according to the United States National Centre for Health Statistics report (2). Recurrence of the disease following surgery can be observed in one-third of the cases and one-fourth of the patients exhibit metastatic disease at the time of a diagnosis (1,2). RCC metastases are often regarded as radioresistant tumors, which was observed in the present case (2–4). For this reason, metastases are usually treated with relatively high biologically effective doses. Metastasis to adjacent organs and bone are common, but distant metastases to the head and neck region are rare. Of these previously reported cases, the facial skin area has been the most common location. The present study demonstrates the case of rapidly growing and radiotherapy-resistant RCC metastasis to the lower lip and chin which was treated with surgery. The functional and esthetic outcome was satisfactory despite the large gap generated by the metastasis resection. This case provides evidence that palliative surgery may achieve a higher quality of life for end-stage oncological patients.

## Case report

**Figure 1:**
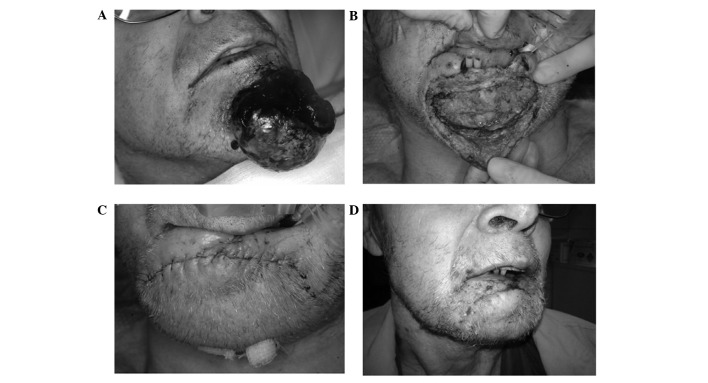
Labial and cutaneous renal cell carcinoma metastasis. (A) The patient exhibited a spontaneously bleeding mass, which had doubled in size within one week. (B) The tumor was resected with 5-mm clinical margins and transcutaneous sutures fixed to the titanium chin plate were used to support the skin and facilitate wound closure. (C) The lip was reconstructed and the skin was suspended by the titanium plate to support lip closure and prevent wound traction. (D) Postoperative follow-up at three weeks indicated no recurrent metastasis.

**Figure 2:**
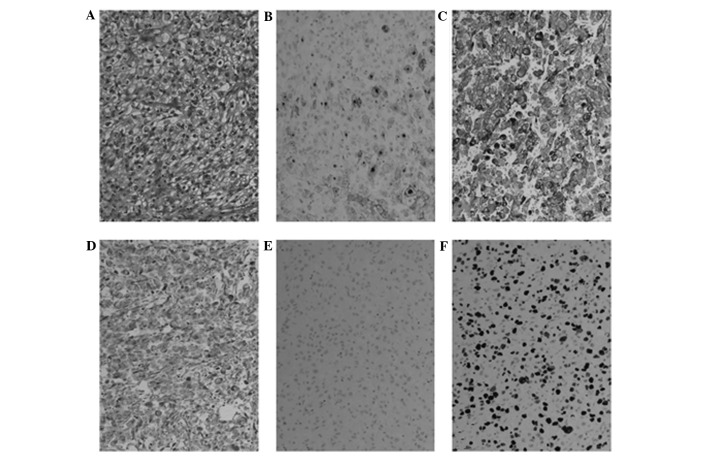
Histological analysis of the mass demonstrated renal cell carcioma metastasis. (A) Hematoxylin and eosin staining identified clear cell differentiation. Immunostaining revealed (B) some positivity for cluster of differentiation 10, in addition to (C) strong positivity for pan-cytokeratin (CK). The tumor was (D) positive for vimentin and (E) negative for CK7. (F) The Mib-1 proliferation index was high and in certain areas increased to ≤70%. Magnification, ×200.

The current study presents the case of a 71-year-old male patient who was diagnosed with RCC in September 2011. At that time, the disease was at an advanced stage. The primary tumor in the lower pool of the right kidney was infiltrating the adjacent structures and the patient exhibited synchronous mediastinal and pleural metastases, with the latter causing persistent pleural effusion and markedly declining lung function. Due to the poor performance status and risk of side effects, the patient refused to initiate the disease-controlling sunitinib treatment and chose to proceed to the optimum supportive care. The patient presented with subcutaneous metastases to the lower lip and back of the neck 11 months after the diagnosis. The patient received palliative radiotherapy (split course, 15/5 Gy) to the rapidly growing lower lip metastasis. The tumor diameter was 1.5 cm when the treatment was initiated. However, no clinical response to radiotherapy was obtained, and three weeks following the treatment the tumor had more than tripled in diameter. Thus, the patient was evaluated at the Department of Oral and Maxillofacial Diseases (Helsinki University; Helsinki, Finland). At the time of admission the patient had a spontaneously bleeding mass (size, 60×60 mm) in the lower lip and the anterior mandible area (Fig. 1A). In addition to this, there was a group of smaller subcutaneous metastases located at the subcutaneous nuchal area, which did not exhibit symptoms. Resection of the lip metastasis was performed with 5-mm clinical margins and for this reason, the resection was extended to the bony surface of the mandible. The lower lip was also partially resected as the small subcutaneous metastases had continued to spread into the lip mucosa (Fig. 1B). To prevent wound tension following closure, the skin was dissected subcutaneously from the resection line to the upper neck, pulled over the chin to cover visible bone, and resuspended with transcutaneous sutures to the titanium plate (MatrixMFACE Plating System; Synthes Holding AG, Solothurn, Switzerland) in the mandible (Fig. 1B and C). The patient was satisfied with the outcome at the three-week postoperative follow-up and no clinical sign of recurrence was observed (Fig. 1D). Histological examination via immunohistochemical staining (Fig. 2) identified the tumor as metastatic RCC and the mass was resected with clear lateral margins.

## Discussion

RCC commonly metastases to adjacent organs, and up to one-fourth of patients have metastases present at the time of the diagnosis (1,2). Four major subtypes of RCC exist (clear-cell, papillary, oncocytoma and collecting duct carcinoma), with different invasion and metastatic potentials. However, none of them have been reported to be particularly invasive to the head and neck region (1,2). Of the 75 previously reported cases of metastatic RCC to the head and neck region, the majority were already diagnosed with RCC, however, certain patients exhibited oral metastasis as the initial manifestation of the disease. This highlights the importance of full body imaging to identify whether the patient has previously undergone surgery for head and neck neoplasms, to avoid inaccurately diagnosing a newly formed metastasis as the recurrence of a former tumor. A third of the previously identified cases of patients with head and neck RCC metastases have been reported on the facial skin area (4–11), although the parotid gland, paranasal sinuses and tongue are also common locations. In addition, single cases of nephroblastoma (also termed, Wilms’ tumor) and renal sarcomas have been reported in the head and neck area (12,13). According to earlier reports, none of the RCC subtypes preferentially metastasize to the head and neck area. The locations of previously reported metastases are listed in Table I (4–51).

In conclusion, surgery is rarely the first option when treating RCC patients with multiple metastases. However, it is important to consider palliative surgery for certain patients, as surgical management of the metastasis may provide an improved quality of life although this type of surgery does not affect the final outcome.

## Figures and Tables

**Table I:** Sites of renal cell carcinoma metastases in the head and neck area obtained from previous studies.

Location	Cases, n (refs)
Skin and subcutaneous lymph nodes	24 (4–11)
Parotid gland	10 (10,18,22,24,33,35,39,41,45)
Tongue	8 (14,15,19,23,31,36,48,51)
Oral mucosa	6 (20,29,30,34,36,40)
Tonsils, facial muscles and oropharynx	9 (21,27,32,34,36,40,50)
Nasal cavity and paranasal sinuses	10 (25,26,28,36,39,42,47,49)
Orbit	3 (15,17,43)
Mandible	3 (12,13,43)
Maxilla	2 (8,16)

The primary location of the renal cell carcinoma was included in the table when various locations were stated in a single study.
